# Liver Disorders in Inflammatory Bowel Disease

**DOI:** 10.1155/2012/642923

**Published:** 2012-02-15

**Authors:** Victor Uko, Suraj Thangada, Kadakkal Radhakrishnan

**Affiliations:** Department of Pediatric Gastroenterology and Hepatology, Cleveland Clinic, Cleveland, OH 44195, USA

## Abstract

Disorders of the hepatobiliary system are relatively common extraintestinal manifestations of inflammatory bowel disease (IBD). These disorders are sometimes due to a shared pathogenesis with IBD as seen in primary sclerosing cholangitis (PSC) and small-duct primary sclerosing cholangitis (small-duct PSC). There are also hepatobiliary manifestations such as cholelithiasis and portal vein thrombosis that occur due to the effects of chronic inflammation and the severity of bowel disease. Lastly, medications used in IBD such as sulfasalazine, thiopurines, and methotrexate can adversely affect the liver. It is important to be cognizant of these disorders as some do have serious long-term consequences. The management of these disorders often requires the expertise of multidisciplinary teams to achieve the best outcomes.

## 1. Introduction

Inflammatory bowel disease (IBD) is a chronic immune-related disorder of the gastrointestinal tract [1]. Disorders of the liver and biliary tract can occur as extraintestinal manifestations of IBD. The clinical course of these disorders does not always correlate with disease activity and can be independent of the degree of intestinal inflammation [2]. The hepatobiliary manifestations in IBD may occur due to a shared pathogenesis such as in primary sclerosing cholangitis (PSC) and small-duct primary sclerosing cholangitis (small-duct PSC). There are also hepatobiliary manifestations such as cholelithiasis and portal vein thrombosis that occur due to the effects of chronic inflammation and the severity of bowel disease (see Table 1). Medications used in IBD such as sulfasalazine, thiopurines, and methotrexate can also have adverse effects on the liver.

The goal of this paper is to highlight the common and some of the less common hepatobiliary manifestations of IBD as these are very important extraintestinal manifestations of the disease.

## 2. Primary Sclerosing Cholangitis (PSC) 

Primary sclerosing cholangitis (PSC) is a chronic fibro-proliferative disorder of the biliary tree that frequently leads to hepatic failure and death in untreated patients [3, 4]. It affects both young and middle-aged patients especially those with underlying inflammatory bowel disease (IBD) [5]. Smith et al. first described the association between PSC and IBD. PSC is the most common hepatobiliary disorder associated with IBD [6, 7]. The natural course of PSC is varied. It typically manifests as a progressive inflammatory state with resultant obliterative fibrosis and destruction of both the intra- and extrahepatic biliary tree. These changes eventually lead to the development of liver cirrhosis and failure [8]. The prevalence of PSC in IBD is such that approximately 70–80% of patients with PSC have IBD. Between 1.4–7.5% of patients with IBD will develop PSC at some point during the course of their disease [9]. The median age of diagnosis of PSC is approximately 40 years of age [3]. In a descriptive study looking at the natural history of PSC and IBD in pediatric patients, the median age of diagnosis of IBD and PSC was 13 and 14 years of age, respectively. This study also showed that the time from the development of symptoms of IBD to the diagnosis of PSC was 2.3 months [4]. There is a male predominance with this disease and a higher prevalence in patients with Ulcerative colitis (UC) as compared to those with Crohn's disease (CD) [3, 4]. The diagnosis of PSC may precede that of IBD but the converse is also known to occur. In the study by Faubion et al, 11% of pediatric patients with PSC had asymptomatic IBD at the time of diagnosis [4]. 

The etiology and pathogenesis of PSC still remains unclear. There have been several genetic factors linked with a susceptibility to this disorder, and these include HLA-B8, HLA-DRB1∗0301(DR3), HLA-DRB3∗0101(DRw52a), and HLA-DRB1∗0401(DR4) [10, 11]. There are also a variety of autoantigens that have been detected in patients with PSC such as antinuclear antibodies (ANA) in 24–53%, smooth muscle antibodies (SMA) in 13–20%, and antiperinuclear cytoplasmic antibody (pANCA) in 65–88% of patients [12–15]. The coexistence of PSC and IBD represents a distinct clinical phenotype as compared with IBD patients without PSC. Studies have shown that patients with coexisting PSC and IBD have a higher incidence of rectal sparing, backwash ileitis, pancolitis, colitis-associated neoplasia, and poorer survival hence the term PSC-IBD [16–18]. The clinical presentation of PSC is often varied, and most patients are asymptomatic at diagnosis. Clinical features of those with symptoms include fatigue, abdominal pain, pruritus, jaundice, and weight loss. The biochemical parameters are consistent with cholestasis and characteristically show a marked elevation of the serum alkaline phosphatase and abnormal liver transaminases [19, 20]. Radiological evaluation is pivotal in the diagnosis of PSC. The demonstration of diffuse, multifocal strictures involving the medium- to large- sized intra- and extrahepatic ductal system forms the basis for the diagnosis. The use of magnetic resonance cholangiopancreaticography (MRCP) provides a sensitive and noninvasive tool for diagnosis. Endoscopic retrograde cholangiopancreaticography (ERCP) is superior in diagnostic accuracy but has an increased risk for procedure-related complications [21–25]. The role of liver biopsies in the diagnosis of PSC is limited and seems to have more relevance in delineating conditions such as small-duct PSC or pericholangitis in patients with IBD. This is thought to be part of a disease spectrum of PSC with features of cholestatic liver disease in the presence of a normal cholangiographic examination [26]. 

Patients with PSC are likely to progress to end-stage liver disease with the attendant complications of portal hypertension such as ascites, varices, and hepatic encephalopathy. Bleeding from varices may present early in the disease due to localized vascular damage in the liver and presinusoidal portal hypertension. Cholangiocarcinoma remains a dreaded complication of PSC. It is not typically seen in the pediatric age group but can arise at any stage of PSC. It has an annual incidence of 0.6–1% and can present as an intraductal tumor or liver mass [27, 28]. Noninvasive tools have been developed to determine disease progression and prognosis in PSC. One such tool is a model developed in The Mayo Clinic. This model estimates the patient survival in PSC using reproducible parameters such as age, serum bilirubin, albumin, aspartate aminotransferase (AST), and the presence of variceal bleeding [29]. The management of PSC in the presence or absence of IBD still poses a significant challenge. Pharmacological interventions have not been shown to alter the progression of the disease. Ursodeoxycholic acid (UDCA) which is often used in the treatment of PSC has been shown to improve the liver enzyme abnormalities with no beneficial change in liver histology or survival without liver transplantation [30]. The largest follow-up series in children with PSC showed that the overall median survival free of liver transplantation was 12.7 years. These patients had a significantly shorter survival than expected for their age- and gender-matched population [31]. The use of UCDA in the treatment of patients with PSC has some benefit in the prevention of colonic neoplasia [32]. The treatment of choice for end-stage PSC or PSC with cholangiocarcinoma is orthotopic liver transplantation (OLT). The 5- and 10- year survival rates following OLT in patients with PSC are 85% and 70%, respectively [33]. The recurrence rate in the transplanted liver is approximately 20–25% [34].

## 3. Small-Duct Primary Sclerosing Cholangitis (Small-Duct PSC)

This is a form of liver disease that is seen in patients with IBD. It was previously termed as pericholangitis. The presence of IBD has been specified by some authors as a prerequisite for the diagnosis of small-duct PSC [35]. These groups of patients are thought to represent a spectrum of PSC. The precise timing of the onset of this disorder in patients with IBD is unclear but it is very similar to PSC. They typically present with features consistent with PSC but have a normal cholangiogram [26, 36]. In a large multicenter study 80% of patients with small-duct PSC had IBD diagnosed at followup or at the time of the diagnosis of liver disease. 78% of these patients had Ulcerative colitis (UC) while 21% had Crohn's colitis [37]. Progression of small duct PSC to PSC can occur in up to 12–23% of patients. There are however no reports of cholangiocarcinoma in this group of patients unless in the setting of disease progression to PSC (large duct) [37]. Some patients may require OLT secondary to progression of the disease; however there remains a risk of recurrence in the transplanted liver. The diagnosis of small duct PSC is best achieved with the aid of a liver biopsy, as these patients characteristically have a normal cholangiographic examination despite the cholestatic clinical picture [26].

## 4. Primary Sclerosing Cholangitis/Autoimmune Hepatitis (PSC/AIH) Overlap Syndrome 

The diagnostic criteria for this condition are not clearly validated. It is however suspected in patients who meet the diagnostic criteria for AIH based on the International Autoimmune Hepatitis Group. The liver histology in these patients shows piecemeal necrosis, rosetting, moderate or severe periportal inflammation, and lymphocyte infiltration [38]. This disorder is well described in patients with IBD especially UC [39]. There is published data that shows patients with IBD and AIH who subsequently go on to develop PSC [38, 40, 41]. The significance of this relationship with IBD is still unclear, and the onset of disease can occur at anytime before or after the diagnosis of IBD. Patients with PSC/AIH however seem to have benefit from treatment with immunosuppressive medications and may have a better prognosis as compared to patients with isolated PSC [40].

## 5. Cholelithiasis

This is a relatively common entity in patients with IBD. It is more prevalent in patients with Crohn's disease (CD) as compared to those with Ulcerative colitis (UC). The incidence of cholelithiasis in patients with CD ranges from 13 to 14% [42–47]. Patients with CD seem to have twice the risk of developing cholelithiasis as compared to IBD-free controls [48]. The factors associated with the increased development of cholelithiasis include location of CD disease at diagnosis, surgery, and extent of ileal resection. Other factors include the age of the patient (uncommon in children), frequency of clinical recurrences, length of hospital stay, and the use of total parenteral nutrition [48]. Patients with CD have been shown to have reduced gallbladder motility which may also lead to the development of cholelithiasis [49, 50]. The formation of cholesterol-supersaturated bile, increased enterohepatic circulation and disruption of bile reabsorption from a diseased terminal ileum, increases the risk for cholelithiasis in patients with CD [43, 51, 52].

## 6. Medication-Associated Liver Disease in Inflammatory Bowel Disease

### 6.1. Sulfasalazine

These class of medications are made up of sulfapyridine (a sulfonamide moiety) linked to 5-aminosalicylic acid (5-ASA) via an azo bond. Sulfasalazine plays an important role in both the induction and maintenance of remission in IBD. Hypersensitivity to sulfasalazine can induce hepatotoxicity which manifests as elevation of liver aminotransferases, hyperbilirubinemia, hepatomegaly, fever, and lymphadenopathy [53, 54]. Studies have shown that the 5-ASA components of the medication can induce both an acute and a chronic hepatitis which can occur from anywhere between 6 days to 1 year after the initiation of treatment, but the frequency of hepatitis is no different between the 5-ASA alone and sulfasalazine [55–59]. 

### 6.2. Thiopurines

Thiopurines (Azathioprine and 6 mercaptopurine) are immunomodulators used in the treatment of IBD. Their mechanism of action is based on conversion to the active metabolite 6-thioguanine [60]. Hepatotoxicity can occur with the use of Azathioprine (AZA) or 6-mercaptopurine (6-MP) due to the effect of another metabolite, 6 methylmercaptopurine (6-MMP) [61–64]. The enzyme that plays a key role in the formation of 6-MMP is thiopurine s-methyl transferase, and as such a better understanding of the genetic heterogeneity of this enzyme may be useful in determining those at risk for hepatotoxicity [61, 64]. The level of 6-MMP in the blood has been measured in children, to identify those at risk for drug-related hepatotoxicity [61]. A much rarer idiosyncratic reaction can occur with 6MP/AZA presenting with features of venoocclusive disease. This occurs due to damage to the sinusoidal endothelial cells and occlusion of the central vein [61, 65–67]. This idiosyncratic reaction can occur at anytime and is independent of the duration of medication usage. Discontinuation of these medications most often leads to reversal of the acute hepatocellular toxicity; however in some instances the development of severe cholestasis may not improve despite withdrawal of the medications [68–70].

### 6.3. Methotrexate

The mechanism of action of this medication is based on its ability to inhibit dihydrofolate reductase, which is an important enzyme in the cell life cycle [71]. The major storage form of methotrexate in the liver is as a polyglutamate metabolite which is potentially hepatotoxic with long-term use [72]. There are no clear guidelines for the monitoring of liver functions in IBD patients on methotrexate, and liver toxicity may become apparent at anytime with the use of this medication. There are however certain risk factors for hepatotoxicity with methotrexate, and these include alcohol use, obesity, diabetes mellitus, abnormal liver enzymes at baseline, and a cumulative dose of greater than 1.5 grams [73].

### 6.4. Biologic Agents

Biologic agents such as Infliximab and Adalimumab have been associated with hepatotoxicity [74]. These medications interfere with the activity of tumor necrosis factor *α* (TNF *α*) which plays a role in hepatocyte regeneration, hence the possible link with the development of hepatotoxicity which is still an uncommon side effect with this group of medications [75, 76].

Patients with chronic hepatitis secondary to hepatitis B or C may have reactivation of the disease and viral replication as a result of immunosuppression from biologic agents [77, 78]. Animal studies have shown that TNF *α* plays an important role in the clearance of hepatitis B from infected hepatocytes by the inhibition of viral replication and promotion of T- cell responses [79, 80]. The concern for viral reactivation is of immense importance as studies have shown a high prevalence of hepatitis B or C in adult patients with CD [81]. Due to the grave implications of reactivation of hepatitis B or C virus in immunosuppressed patients, guidelines have been proposed for the routine screening of patients for these viruses before starting biologic agents [82].

## 7. Less Common Liver Disorders in Inflammatory Bowel Disease

### 7.1. Portal Vein Thrombosis

This is a relatively rare but significant thromboembolic complication of IBD. Patients with IBD are at risk for the development of venous thrombosis. Patients with IBD also have elevated platelet counts, fibrinogen, factor V and VIII levels. There is also a concomitant decrease in antithrombin III levels. The presence of chronic bowel inflammation is also a risk factor for thrombosis in these patients [83]. Portal vein thrombosis is more likely to occur in the postoperative setting and has been reported in patients with UC after restorative proctocolectomy [84–87].

### 7.2. Budd Chiari Syndrome

There are published case reports of Budd Chiari syndrome occurring in patients with IBD [88, 89]. This is a thromboembolic phenomenon that can occur at baseline in patients with UC but in the setting of an acute flare the risk is up to eight times higher [90]. The perioperative period is also a significant risk period for thromboembolism.

### 7.3. Fatty Liver

Sonographic evidence of fatty liver is not an uncommon finding in patients with IBD with a prevalence of up to 35% in a recent study of 511 IBD patients [46]. Patients with fatty liver often have no symptoms related to the liver; however there is reported correlation between the severity of colitis and fatty liver changes in patients with IBD especially UC [91]. The timing of the onset of fatty liver in patients with IBD is still unclear. Current therapy is targeted towards the treatment of the bowel disease and improving the overall nutritional status of the patient.

### 7.4. Granulomatous Hepatitis

This is a rare occurrence found on liver histology in patients with IBD and is usually seen secondary to treatment with sulfasalazine. It is said to occur in less than 1% of patients [92]. It can also be seen with other non-IBD conditions such as malignancies or infections.

## 8. Conclusion

Hepatobiliary manifestations do occur in IBD and may have various etiopathogenetic origins. These disorders vary with regard to the degree of severity ranging from the more complex disorders such as PSC to the potentially less severe manifestation such as cholelithiasis. It is however of critical importance to remain vigilant for subtle manifestations of these disorders so as to institute appropriate care which may require an interdisciplinary approach involving multiple subspecialty teams such as general surgery, colorectal surgery, and transplant surgery. 

## Figures and Tables

**Table 1:** Liver disorders in inflammatory bowel disease.

	Ulcerative colitis	Crohn's disease
More common	Primary sclerosing cholangitis (PSC)	Gallstones
	Small-duct PSC	Medication-associated liver disease
	Autoimmune hepatitis/PSC overlap	Fatty liver
	Medication-associated liver disease	
	Fatty liver	

Less common	Gallstones	Primary sclerosing cholangitis (PSC)
		Small-duct PSC

Rare	Portal vein thrombosis	Portal vein thrombosis
	Liver abscess	Liver abscess
	Granulomatous hepatitis	Granulomatous hepatitis

## Abbreviations

IBD: Inflammatory bowel disease,
CD: Crohn's disease,
UC: Ulcerative colitis,
PSC: Primary sclerosing cholangitis,
AIH: Autoimmune hepatitis,
ERCP: Endoscopic retrograde cholangiopancreaticography,
MRCP: Magnetic resonance cholangiopancreaticography,
UCDA: Ursodeoxycholic acid,
